# A real-world comparison of tisagenlecleucel and axicabtagene ciloleucel CAR T cells in relapsed or refractory diffuse large B cell lymphoma

**DOI:** 10.1038/s41591-022-01969-y

**Published:** 2022-09-22

**Authors:** Emmanuel Bachy, Steven Le Gouill, Roberta Di Blasi, Pierre Sesques, Guillaume Manson, Guillaume Cartron, David Beauvais, Louise Roulin, François Xavier Gros, Marie Thérèse Rubio, Pierre Bories, Jacques Olivier Bay, Cristina Castilla Llorente, Sylvain Choquet, René-Olivier Casasnovas, Mohamad Mohty, Stéphanie Guidez, Magalie Joris, Michaël Loschi, Sylvain Carras, Julie Abraham, Adrien Chauchet, Laurianne Drieu La Rochelle, Bénédicte Deau-Fischer, Olivier Hermine, Thomas Gastinne, Jean Jacques Tudesq, Elodie Gat, Florence Broussais, Catherine Thieblemont, Roch Houot, Franck Morschhauser

**Affiliations:** 1 Hematology Department, Hospices Civils de Lyon, Pierre Bénite, Lyon, France; 2 International Center for Infectiology Research (CIRI), Inserm U1111, Lyon, France; 3 Hematology Department, Institut Curie, Paris, France; 4 Hematology Department, Hôpital Saint Louis, Paris, France; 5 Hematology Department, CHU de Rennes, Rennes, France; 6 Hematology Department, CHU de Montpellier & UMR-CNRS, Montpellier, France; 7 Hematology Department, CHU de Lille, Lille, France; 8 Hematology Department, Hôpital Henri Mondor, Créteil, France; 9 Hematology Department, CHU de Bordeaux, Bordeaux, France; 10 Hematology Department, CHU de Nancy, Nancy, France; 11 Hematology Department, CHU de Toulouse, Toulouse, France; 12 Hematology Department, CHU de Clermont Ferrand, Clermont-Ferrand, France; 13 Hematology Department, Gustave Roussy Cancer Campus, Villejuif, Paris, France; 14 Hematology Department, Hôpital de la Pitié Salpêtrière & AP-HP Sorbonne Université, Paris, France; 15 Hematology Department, CHU de Dijon, Dijon, France; 16 Hematology Department, Hôpital Saint Antoine & Sorbonne University & Inserm UMRs 938, Paris, France; 17 Hematology Department, CHU de Poitiers, Poitiers, France; 18 Hematology Department, CHU d’Amiens, Amiens, France; 19 Hematology Department, CHU de Nice, Nice, France; 20 Hematology Department, CHU de Grenoble & University Grenoble-Alpes, Institute for Advanced Biosciences, La Tronche, France; 21 Hematology Department, CHU de Limoges, Limoges, France; 22 Hematology Department, CHU de Besançon, Besançon, France; 23 Hematology Department, CHU de Tours, Tours, France; 24 Hematology Department, Hôpital Cochin, Paris, France; 25 Hematology Department, Hôpital Necker, Paris, France; 26 Hematology Department, CHU de Nantes, Nantes, France; 27 Biostatistics Department, LYSARC, Lyon, France; 28 Medical and Scientific Affairs Department, LYSARC, Lyon, France; 29 Lille University, ULR 7365 - GRITA - Groupe de Recherche sur les formes Injectables et les Technologies Associées, Lille, France

**Keywords:** Outcomes research, B-cell lymphoma, Cancer immunotherapy

## Abstract

Axicabtagene ciloleucel (axi-cel) and tisagenlecleucel (tisa-cel) have both demonstrated impressive clinical activity in relapsed/refractory (R/R) diffuse large B cell lymphoma (DLBCL). In this study, we analyzed the outcome of 809 patients with R/R DLBCL after two or more previous lines of treatment who had a commercial chimeric antigen receptor (CAR) T cells order for axi-cel or tisa-cel and were registered in the retrospective French DESCAR-T registry study (NCT04328298). After 1:1 propensity score matching (*n* = 418), the best overall response rate/complete response rate (ORR/CRR) was 80%/60% versus 66%/42% for patients treated with axi-cel compared to tisa-cel, respectively (*P* < 0.001 for both ORR and CRR comparisons). After a median follow-up of 11.7 months, the 1-year progression-free survival was 46.6% for axi-cel and 33.2% for tisa-cel (hazard ratio (HR) = 0.61; 95% confidence interval (CI), 0.46–0.79; *P* = 0.0003). Overall survival (OS) was also significantly improved after axi-cel infusion compared to after tisa-cel infusion (1-year OS 63.5% versus 48.8%; HR = 0.63; 95% CI, 0.45–0.88; *P* = 0.0072). Similar findings were observed using the inverse probability of treatment weighting statistical approach. Grade 1–2 cytokine release syndrome was significantly more frequent with axi-cel than with tisa-cel, but no significant difference was observed for grade ≥3. Regarding immune effector cell-associated neurotoxicity syndrome (ICANS), both grade 1–2 and grade ≥3 ICANS were significantly more frequent with axi-cel than with tisa-cel. In conclusion, our matched comparison study supports a higher efficacy and also a higher toxicity of axi-cel compared to tisa-cel in the third or more treatment line for R/R DLBCL.

## Abstract (web summary)

Analysis of outcomes of over 800 patients with relapsed/refractory diffuse large B cell lymphoma, treated with commercially available CAR T cell therapy, supports higher efficacy and also a higher toxicity of axicabtagene ciloleucel compared to tisagenlecleucel as the third or more treatment line for this type of tumor.

## Main

DLBCL is the most common lymphoma subtype, accounting for about 40% of all non-Hodgkin lymphomas^1^. CAR T cell therapies targeting CD19 have shown impressive efficacy and manageable toxicity for the treatment of various lymphoma histology subtypes, such as mantle cell lymphoma, follicular lymphoma and DLBCL^2–7^. Tisagenlecleucel (tisa-cel) and axicabtagene ciloleucel (axi-cel) are two CAR T products that were initially approved for the treatment of DLBCL in the third or subsequent line of treatment. Tisa-cel is a 4-1BB co-stimulatory domain-based second-generation CAR T, whereas axi-cel is CD28 based. Approvals were granted after the results of the JULIET and ZUMA-1 pivotal studies demonstrating best ORR/CRR of 52%/40% and 82%/58% for tisa-cel and axi-cel, respectively^5,6,8,9^. The recent updated follow-up of ZUMA-1 after 5 years suggested that ~40% of patients might be cured with CAR T in this setting^10^. In the last 2 years, many publications based on real-life data from various countries worldwide have confirmed the high response rates, prolonged response duration and survival achieved with CAR T in DLBCL^11–15^. Strikingly, and despite stringent patient selection in clinical trials, efficacy in the non-trial setting seems to parallel results obtained in pivotal studies, and toxicity appears significantly lower in real life due to the earlier mitigating strategy with anti-interleukin-6 and steroids use^16,17^. A multitude of parameters can impact efficacy and safety of CAR T, such as, among many others, the use of a bridging therapy to control for disease progression during product manufacturing, the tumor bulk or the delay between leukapheresis and infusion^18,19^. Therefore, the need for real-world evidence (RWE) studies to apprehend this fast-moving field has never been so high.

Crude response rates and safety reports from clinical trials suggest higher efficacy and toxicity associated with the use of axi-cel compared to tisa-cel^5,6^. However, these conclusions might be misleading due to large differences between study designs: (1) patients with primary mediastinal B cell lymphoma (PMBCL) were enrolled in ZUMA-1 but not in JULIET; (2) the doses of fludarabine and cyclophosphamide as conditioning regimen were higher in ZUMA-1; and (3) bridging chemotherapy to control for disease progression during the CAR T manufacturing process was allowed in JULIET but not in ZUMA-1 (refs. ^5,6^). The latter introduced a major bias precluding any possible direct comparison between studies because patients with more aggressive lymphomas cannot usually be spared from bridging therapy between leukapheresis and lymphodepletion.

Several matching-adjusted indirect comparisons (MAICs) have been attempted to compare different CAR T products^20,21^. MAIC uses individual patient data (IPD) from one study and trial-level data from another to form a population-adjusted indirect comparison between treatments. One of these recently reported MAICs suggests that axi-cel is superior to tisa-cel for disease control but is associated with significantly more toxicity^20^. In addition, despite increasing popularity, many biases remain with such statistical methods^22–24^.

Since 2019, the French Health Authorities have required extensive data collection for each patient with a theoretical indication of CAR T treatment. Reimbursement is conditional on data comprehensive completion by the local investigator. The DESCAR-T registry has been set up by the Lymphoma Study Association (LYSA) and the Lymphoma Academic Research Organization (LYSARC) to fulfil this regulatory request and to allow for comprehensive RWE studies.

Given the lack of an adequate comparison for efficacy and safety between tisa-cel and axi-cel, we embarked on an IPD-based matched comparison considering all French patients with DLBCL treated with commercial CAR T and included in the DESCAR-T registry.

## Results

### Patient characteristics and outcome

**Fig. 1:**
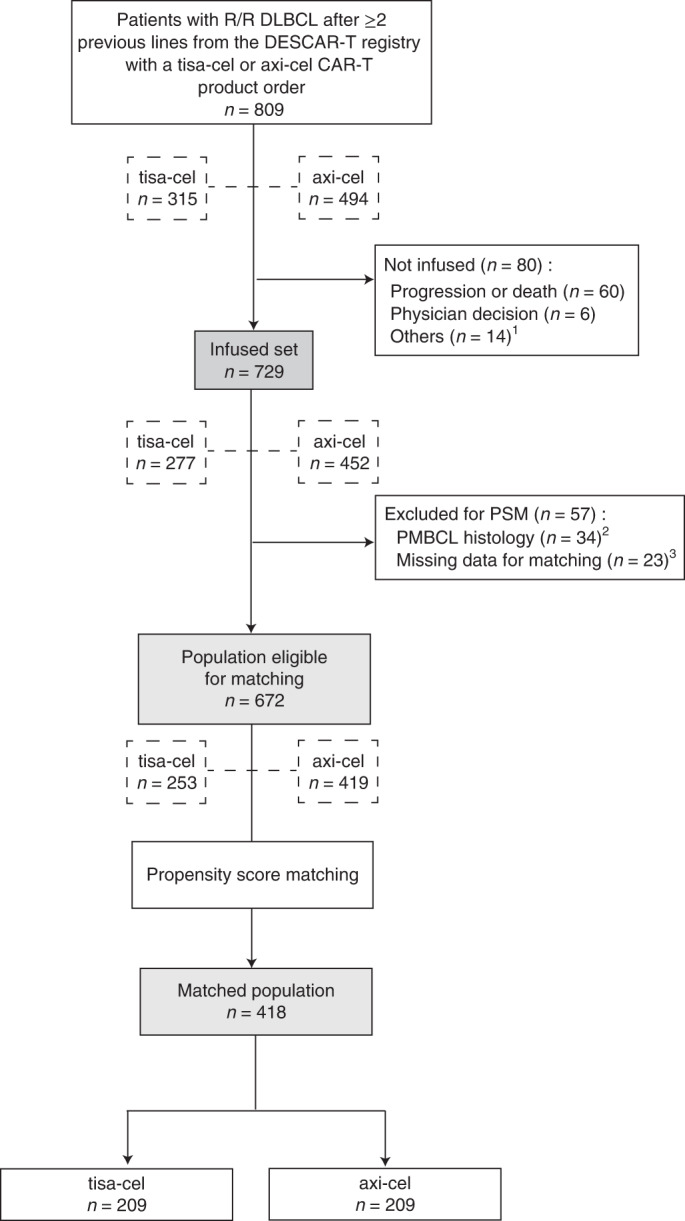
Patient flow diagram for PSM analysis. ^1^Manufacturing failure (*n* = 3), uncontrolled infection (*n* = 3), waiting for infusion (*n* = 3), patient decision (*n* = 1), leukapheresis failure (*n* = 1), acute coronary syndrome (*n* = 1), concomitant malignancy (*n* = 1) and progression of another malignancy (*n* = 1). ^2^Patients with PMBCL histology were excluded because tisa-cel has no approval for this histology. ^3^Patients with ≥25% of missing data for matching covariates were removed from the matching step.

**Fig. 2:**
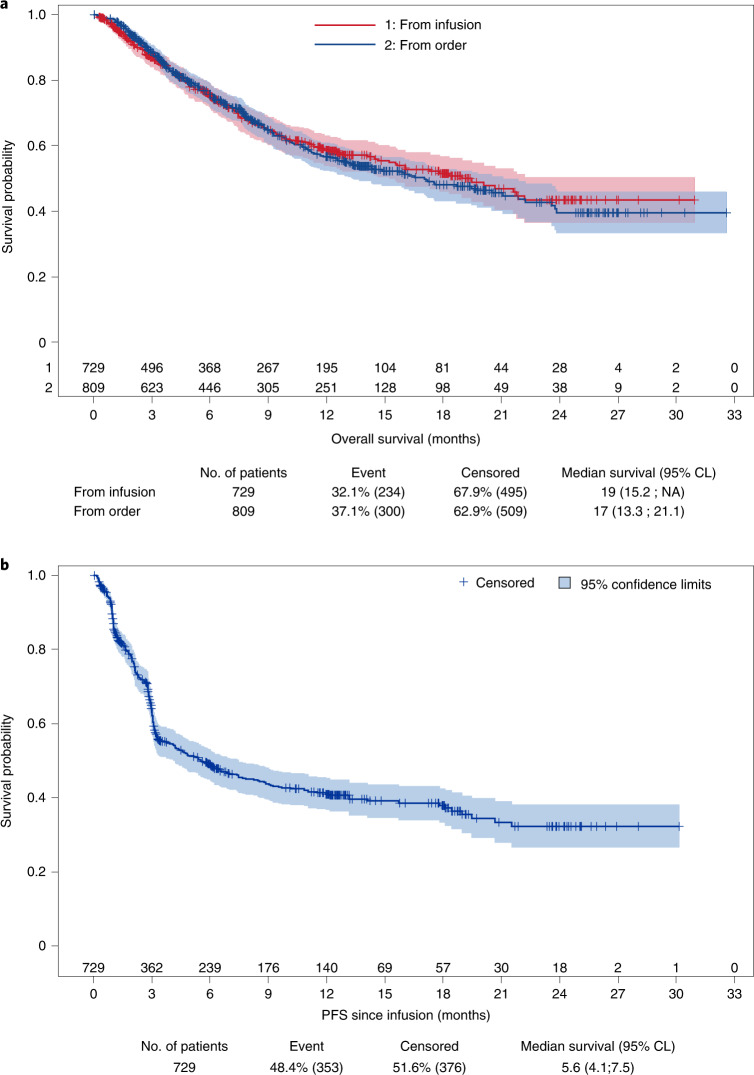
Survival of the whole cohort of patients treated with commercial tisa-cel or axi-cel from the French DESCAR-T registry before any matching. **a**, OS since CAR T order (*n* = 809, blue line) or since CAR T infusion (*n* = 729, red line). For 80 patients, a CAR T product was ordered, but patients did not proceed until infusion due to disease progression or death, physician decision or other reasons (see patient flowchart). **b**, PFS from CAR T infusion (*n* = 729). Shaded areas correspond to the 95% confidence bands using the Hall–Wellner method. CL, confidence limit.

Between December 2019 and October 2021, 809 patients from 23 French centers with R/R DLBCL after at least two lines of previous therapy had a commercial CAR T order for axi-cel or tisa-cel and were registered in DESCAR-T (Fig. 1). Patient characteristics are presented in Table 1. Median age was 63 years (range, 19–81 years), and 61% of patients were male. Median number of prior lines of treatment was three (range, 2–10), and 21% of patients had received a prior stem cell transplant (SCT). The median time between the end date of last treatment and CAR T order was 35 days (Q1;Q3, 15;78 days). Most patients﻿ (*n* = 604, 75%) had DLBCL not otherwise specified (NOS) or high-grade B cell lymphoma (HGBCL); 127 patients (16%) had transformed follicular lymphoma (tFL); 35 patients (4%) had primary mediastinal large B cell lymphoma (PMBCL); and 24 patients (3%) had transformed marginal zone lymphoma (tMZL). Few patients (*n* = 19; 2%) had other histologies (T cell/histiocyte-rich large B cell lymphoma (T/HRLBCL) in 11 patients; systemic relapse of primary central nervous system lymphoma (PCNSL) in four patients; and DLBCL, leg type, in four patients) (Table 1). With a median follow-up of 13 months (95% CI, 12.1–13.5 months), projected median OS was 17 months (95% CI, 13.3–21.1 months) from CAR T order (Fig. 2a).

**Table 1:** Patient characteristics

	All patients^*^				Before PSM^*&^				After PSM^*^			
	Order set		Infusion set		axi-cel		tisa-cel		axi-cel		tisa-cel	
	*n* = 809		*n* = 729		*n* = 419		*n* = 253		*n* = 209		*n* = 209	
Age at time of CAR T order (years)												
Median (min;max)	63 (19;81)		63 (19;81)		63 (19;79)		64 (20;81)		62 (20;79)		64 (20;81)	
Missing	1		1		0		0		0		0	
Sex												
Male	490	(60.6%)	437	(59.9%)	251	(59.9%)	157	(62.1%)	121	(57.9%)	126	(60.3%)
Female	318	(39.3%)	291	(39.9%)	168	(40.1%)	96	(37.9%)	88	(42.1%)	83	(39.7%)
Missing	1	(0.1%)	1	(0.1%)	0	(0.0%)	0	(0.0%)	0	(0.0%)	0	(0.0%)
aaIPI												
0	54	(6.7%)	52	(7.1%)	31	(7.4%)	18	(7.1%)	17	(8.1%)	16	(7.7%)
1	237	(29.3%)	224	(30.7%)	135	(32.2%)	69	(27.3%)	71	(34.0%)	56	(26.8%)
2	373	(46.1%)	336	(46.1%)	190	(45.3%)	126	(49.8%)	89	(42.6%)	105	(50.2%)
3	58	(7.2%)	40	(5.5%)	19	(4.5%)	20	(7.9%)	11	(5.3%)	16	(7.7%)
Missing	87	(10.8%)	77	(10.6%)	44	(10.5%)	20	(7.9%)	21	(10.0%)	16	(7.7%)
ECOG PS												
0–1	665	(82.2%)	613	(84.1%)	361	(86.2%)	208	(82.2%)	178	(85.2%)	173	(82.8%)
≥2	97	(12.0%)	75	(10.3%)	39	(9.3%)	33	(13.0%)	20	(9.6%)	27	(12.9%)
Missing	47	(5.8%)	41	(5.6%)	19	(4.5%)	12	(4.7%)	11	(5.3%)	9	(4.3%)
CRP^†^												
≤30 mg L^−1^	-		521	(71.5%)	313	(74.7%)	175	(69.2%)	150	(71.8%)	147	(70.3%)
>30 mg L^−1^			165	(22.6%)	92	(22.0%)	65	(25.7%)	49	(23.4%)	55	(26.3%)
Missing			43	(5.9%)	14	(3.3%)	13	(5.1%)	10	(4.8%)	7	(3.3%)
LDH^†^												
≤ULN	-		311	(42.7%)	174	(41.5%)	116	(45.8%)	85	(40.7%)	83	(39.7%)
[ULN; 2× ULN]			286	(39.2%)	177	(42.2%)	96	(37.9%)	85	(40.7%)	88	(42.1%)
>2× ULN			87	(11.9%)	50	(11.9%)	30	(11.9%)	30	(14.4%)	29	(13.9%)
Missing			45	(6.2%)	18	(4.3%)	11	(4.3%)	9	(4.3%)	9	(4.3%)
Bulk (with a cutoff at 5 cm)^†^												
No	-		551	(75.6%)	326	(77.8%)	198	(78.3%)	168	(80.4%)	160	(76.6%)
Yes			150	(20.6%)	85	(20.3%)	51	(20.2%)	39	(18.7%)	45	(21.5%)
Missing			28	(3.8%)	8	(1.9%)	4	(1.6%)	2	(1.0%)	4	(1.9%)
Ann Arbor stage												
I	57	(7.0%)	55	(7.5%)	31	(7.4%)	16	(6.3%)	18	(8.6%)	16	(7.7%)
II	90	(11.1%)	85	(11.7%)	51	(12.2%)	25	(9.9%)	26	(12.4%)	22	(10.5%)
III	100	(12.4%)	92	(12.6%)	63	(15.0%)	25	(9.9%)	29	(13.9%)	24	(11.5%)
IV	513	(63.4%)	453	(62.1%)	249	(59.4%)	180	(71.1%)	126	(60.3%)	140	(67.0%)
Missing	49	(6.1%)	44	(6.0%)	25	(6.0%)	7	(2.8%)	10	(4.8%)	7	(3.3%)
Number of prior treatment lines												
Median (min;max)	3 (2;10)		3 (2;10)		3 (2;9)		3 (2;10)		2 (2;8)		2 (2;10)	
Missing	10		9		0		0		0		0	
At least one prior transplant												
No	640	(79.1%)	567	(77.8%)	332	(79.2%)	187	(73.9%)	160	(76.6%)	163	(78.0%)
Yes	169	(20.9%)	162	(22.2%)	87	(20.8%)	66	(26.1%)	49	(23.4%)	46	(22.0%)
Missing	0		0		0		0		0		0	
Time between first CAR T order of center and CAR T order of patient (days)^ø^												
Median (Q1;Q3)	446 (214;681)		446 (206;671)		420 (169;681)		485 (316;662)		517 (174;724)		495 (317;664)	
Missing	0		0		0		0		0		0	
Time between end of last treatment and CAR T infusion (days)^§^												
Median (Q1;Q3)	35 (15;78)		87 (66;138)		90 (68;146)		87 (66;133)		91 (71;132)		92 (68;147)	
Missing	17		16		0		0		0		0	
Bridging and response to bridging												
No bridging	NA		126	(17.3%)	76	(18.1%)	35	(13.8%)	26	(12.4%)	29	(13.9%)
Response to bridging (PR or CR)			188	(25.8%)	105	(25.1%)	72	(28.5%)	65	(31.1%)	57	(27.3%)
No response to bridging (SD or PD)			386	(52.9%)	221	(52.7%)	138	(54.5%)	111	(53.1%)	117	(56.0%)
Missing			29	(4.0%)	17	(4.1%)	8	(3.2%)	7	(3.3%)	6	(2.9%)
Histological diagnosis												
DLBCL NOS or HGBCL	604	(74.7%)	542	(74.3%)	328	(78.2%)	193	(76.3%)	165	(78.9%)	166	(79.4%)
T/HRLBCL	11	(1.3%)	10	(1.4%)	7	(1.7%)	3	(1.2%)	1	(0.5%)	2	(1.0%)
DLBCL after PCNSL	4	(0.5%)	4	(0.5%)	1	(0.2%)	3	(1.2%)	0	(0.0%)	1	(0.5%)
DLBCL, leg type	4	(0.5%)	4	(0.5%)	2	(0.5%)	2	(0.8%)	1	(0.5%)	0	(0.0%)
PMBCL^¶^	35	(4.3%)	34	(4.7%)	NA		NA		NA		NA	
tFL	127	(15.7%)	117	(16.0%)	71	(16.9%)	44	(17.4%)	37	(17.7%)	33	(15.8%)
tMZL	24	(3.0%)	18	(2.5%)	10	(2.4%)	8	(3.2%)	5	(2.4%)	7	(3.3%)
Missing	0		0		0		0		0		0	

^*^Sum may not equal 100% because of rounding.

^†^CRP, LDH and bulk were assessed at time of lymphodepletion.

^§^Except for the order set where time between the last treatment and the CAR T order was considered.

^¶^PMBCL was not considered for PSM because tisa-cel is not approved for this histology.

^ø^Time between first CAR T order of center and CAR T order of patient was used as a surrogate for center experience for CAR T therapy for each given patient.

^&^Patients from the infusion set with more than 25% of missing data and with PMBCL were excluded for the matching procedure.

CR, complete response; NA, not applicable; Q1, first quartile; Q3, third quartile; PD, progressive disease; PR, partial response; SD, stable disease.

**Extended Data Fig. 1:**
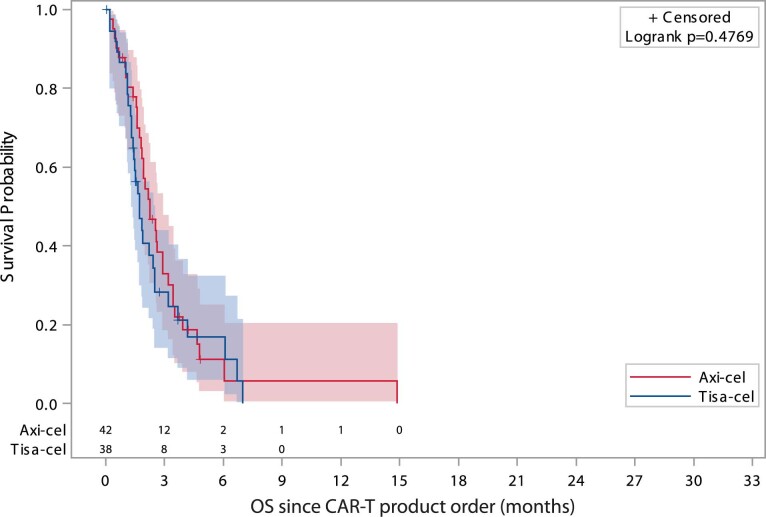
Overall survial (OS) for patients with a CAR-T product order who did not proceed until infusion according to CAR-T type. Extended Data Fig. 1 For 80 patients, a CAR-T product was ordered but was never infused (*n* = 42 for axi-cel, *n* = 38 for tisa-cel) due to disease progression (*n* = 60), physician decision (*n* = 6) and other reasons detailed in the patient flow diagram in Fig. 1 (*n* = 14). Shaded areas correspond to the 95% confidence bands using Hall-Wellner method. *P* value was calculated using a two-sided logrank test.

Sixty patients out of 809 with a CAR T product order progressed or died between leukapheresis and lymphodepletion, and 20 did not proceed to lymphodepletion after physician decision or for other reasons (Fig. 1). Of these 80 patients with a CAR T product order who did not proceed until an infusion (*n* = 38 for tisa-cel and *n* = 42 for axi-cel), OS was expectedly very poor and similar according to CAR T product (axi-cel or tisa-cel) (*P* = 0.48; Extended Data Fig. 1). Finally, 729 patients proceeded to lymphodepletion and CAR T infusion. Characteristics of this patient subpopulation are presented in Table 1. Median OS from infusion was 19.0 months (95% CI, 15.2–not reached), and median progression-free survival (PFS) was 5.6 months (95% CI, 4.1–7.5 months) (Fig. 2a,b).

### Propensity score matching

**Fig. 3:**
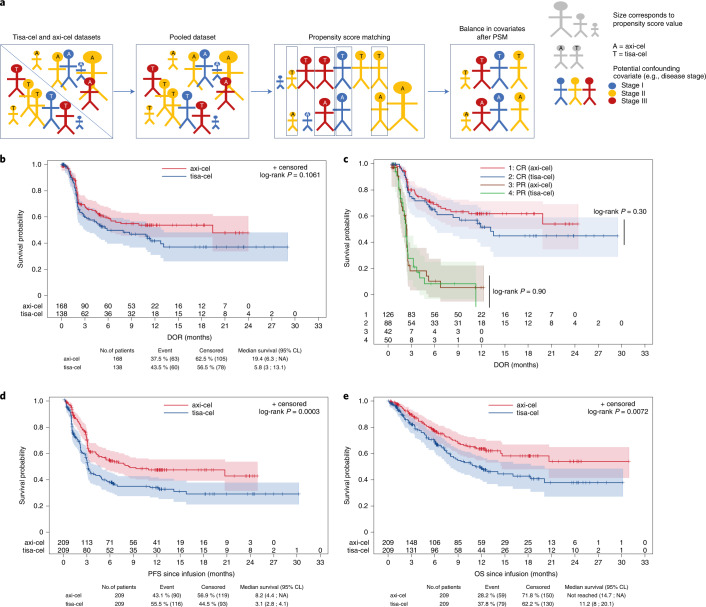
Survival according to CAR T product after PSM. **a**, Propensity score reflects the probability of receiving tisa-cel or axi-cel conditional on an exhaustive list of 14 pre-infusion covariates. PSM is based on matching patients with similar propensity score. Comparability according to each covariate (for instance, disease stage depicted here) of the resulting two sub-cohorts of patients receiving one CAR T or the other is checked using SMDs (Extended Data Fig. 3). **b**, DOR according to CAR T product (axi-cel, *n* = 168, red line; tisa-cel, *n* = 138, blue line) (*P* = 0.11). **c**, DOR according to CAR T product and response quality (complete response (CR) versus partial response (PR); *P* = 0.30 and *P* = 0.90, respectively) (axi-cel and CR, *n* = 126, red line; tisa-cel and CR, *n* = 88, blue line; axi-cel and PR, *n* = 42, brown line; tisa-cel and PR, *n* = 50, green line). **d**, PFS according to CAR T product (*P* = 0.0003). **e**, OS according to CAR T product (*P* = 0.0072). *P* values were calculated using two-sided log-rank tests. No adjustment was made for multiple comparisons. Shaded areas correspond to the 95% confidence bands using the Hall–Wellner method. CL, confidence limit; NA, not assessable.

**Extended Data Fig. 2:**
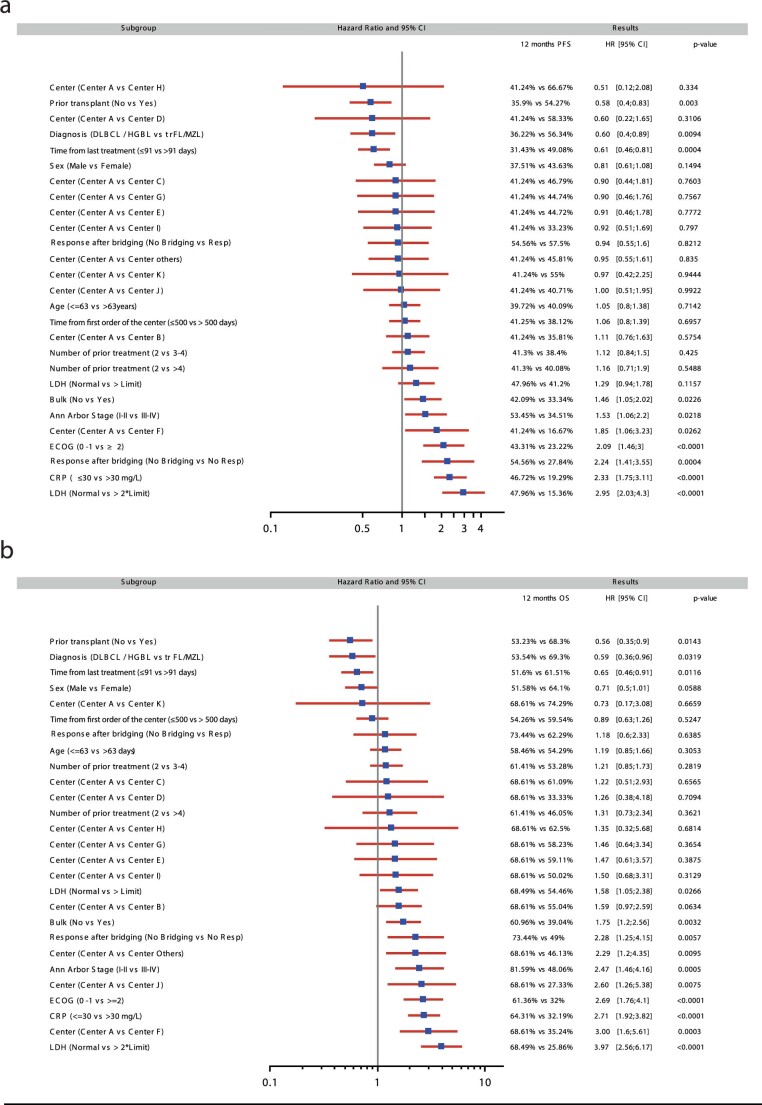
Univariate prognostic analysis. **a**, univariate analysis for progression-free survival (PFS). **b**, univariate analysis for overall survival (OS). Blue point represents the value of the hazard ratio (HR) and red segment the value of the 95% confidence interval (CI). The first category in parentheses is taken as the reference category for comparison and HR computation. For instance, for PFS analysis HR is 2.95 for patients with a LDH level twice above the upper limit of the normal (ULN) compared to patients with a normal value. A HR < 1 represents a prognosis factor associated with a prolonged survival while a HR > 1 represents a prognosis factor associated with a shorter survival. A prognostic factor is statistically significant if the 95% CI does not contain 1. Cox univariate model was used for calculating HR and associated two-sided *P* value. No adjustment was performed for multiple comparisons. All centers were anonymized. Time from last treatment, age and time to first order of the center were dichotomized according to the median value of data distribution. Number of prior treatment, LDH level and bridging were divided into 3 categories (2 vs 3-4 vs >4 prior lines; normal LDH, LDH between 1 and 2 times the ULN and LDH above 2 times the ULN; no bridging vs response to bridging vs no response to bridging, respectively). *Time from last treatment* represents the time from the start of the last treatment to the time of CAR T infusion. *Time from first order of the center* represents the time from the order of the first CAR-T in the center to the time of infusion of the CAR T for the patient (as a surrogate of the “center experience” for CAR-T therapy for a given patient). DLBCL, diffuse large B-cell lymphoma; HGBL, high grade B-cell lymphoma; trFL/MZL, transformed follicular lymphoma or marginal zone lymphoma; Resp; complete or partial response to bridging; No Resp, no response (that is stable or progressive disease) to bridging; ECOG, Eastern Collaborative Oncology Group Performance Status; CRP, C reactive protein; LDH, lactate dehydrogenase.

**Extended Data Fig. 3:**
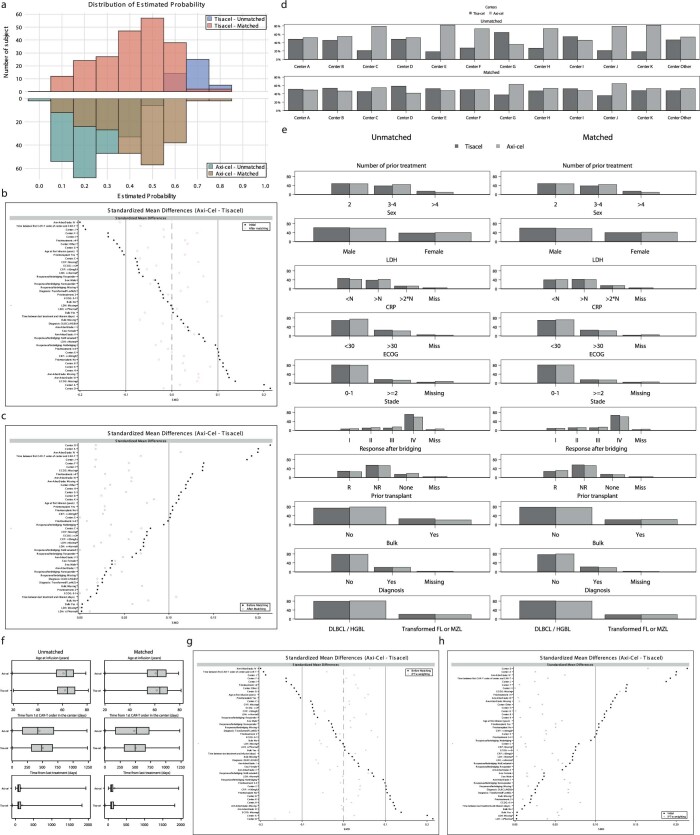
Balance assessment before and after matching. **a**, Propensity score (PS) distribution before and after PSM. **b**, Standardized Mean Differences (SMD) of covariates categories before and after PSM. **c**, Absolute SMD before and after PSM. **d**, CAR T products distribution across centers from the DESCAR-T registry before and after PSM (light grey= axi-cel; dark grey=tisa-cel). **e**, CAR T products distribution according to categorical covariates before and after PSM (light grey= axi-cel; dark grey=tisa-cel). **f**, Balance assessment according to CAR T product for continuous covariates before and after PSM. Box plot represents 1^st^ quartile and 3^rd^ quartile. Line in the middle of the box represents the median. Round symbol represents the mean. Left segment represents distribution from the minimal value. Right segment represents distribution to the maximal value. **g**, SMD of covariates categories before and after IPTW. b, absolute SMD before and after IPTW.

A propensity score is the conditional probability that a patient receives one treatment or another given a set of observed covariates. The aim of propensity score matching (PSM) was to balance covariates between axi-cel and tisa-cel groups to account for all possible measured confounding variables (that is, variables that have a causal relationship with both the measured outcome and the CAR T product used) (Fig. 3a). For PSM, of 729 patients infused with a CAR T product, 34 patients with PMBCL (for which tisa-cel is not approved) and 23 patients with more than 25% of missing data for matching variables were removed before matching (Fig. 1). The final population for matching comprised 253 patients treated with tisa-cel and 419 patients treated with axi-cel. Patient characteristics according to CAR T product are detailed in Table 1. Univariate prognostic analyses for PFS and OS confirmed that many patient characteristics were significantly associated with outcome and were potential confounders when comparing efficacy of CAR T products (Extended Data Fig. 2). After stringent PSM on 14 parameters (Extended Data Fig. 3a), absolute values of the standardized mean differences (SMDs) were less than 0.1 for almost all matching covariates (Extended Data Fig. 3b,c). PSM resulted in a much balanced distribution of CAR T product use across centers (Extended Data Fig. 3d) and according to individual covariates (Extended Data Fig. 3e,f). However, disease severity was still slightly higher for patients treated with tisa-cel than with axi-cel, as exemplified by a higher age-adjusted international prognostic index (aaIPI) score of 2 or 3 (57.9% versus 47.9%). In the 1:1 matched population (*n* = 418; 209 patients treated with axi-cel and 209 patients treated with tisa-cel), the best ORR/CRR was 80.4%/60.3% versus 66.0%/42.1% for patients treated with axi-cel compared to tisa-cel, respectively (*P* < 0.001 for both ORR and CRR comparisons; Table 2). After a median follow-up of 11.7 months (95% CI, 10.5–12.0 months), the duration of response (DOR) was not significantly different between axi-cel and tisa-cel (1-year DOR 53.8% for axi-cel compared to 41.8% for tisa-cel, *P* = 0.106; Fig. 3b). There was no further significant difference in DOR according to the quality of response (complete versus partial) (Fig. 3c). However, the 1-year PFS was 46.6% for axi-cel and 33.2% for tisa-cel (HR = 0.61; 95% CI, 0.46–0.79; *P* = 0.0003; Fig. 3d and Table 2). OS was also significantly improved after axi-cel infusion compared to after tisa-cel infusion (1-year OS 63.5% versus 48.8%; HR = 0.63; 95% CI, 0.45–0.88; *P* = 0.0072; Fig. 3e and Table 2).

**Table 2:** Response rates and survival according to CAR T product in matched populations using PSM and IPTW approaches^a^

	PSM			IPTW		
	axi-cel *n* = 209	tisa-cel *n* = 209	*P*	axi-cel	tisa-cel	*P*
Response rate						
ORR% (95% CI)	80.4 (74.3–85.5)	66.0 (59.2–72.4)	<0.001	78.5 (75.3–81.6)	62.8 (59.2–66.3)	<0.001
CRR% (95% CI)	60.3 (53.3–67.0)	42.1 (35.3–49.1)	<0.001	60.1 (56.4–63.8)	42.0 (38.3–45.6)	<0.001
Survival						
PFS% at 1 year (95% CI)	46.6 (38.5–54.3)	33.2 (25.7–40.8)	0.0003	44.5 (38.7–50.1)	34.7 (26.2–43.3)	0.0005
HR (95% CI)	0.61 (0.46–0.79)	1 (ref)		0.66 (0.53–0.82)	1 (ref)	
DOR% at 1 year (95% CI)	53.8 (44.7–62.1)	41.8 (31.3–51.9)	0.106	51.0 (44.4–57.1)	46.0 (33.0–58.0)	0.482
HR (95% CI)	0.75 (0.53–1.06)	1 (ref)		0.81 (0.60–1.08)	1 (ref)	
OS% at 1 year (95% CI)	63.5 (55.0–70.8)	48.8 (39.7–57.2)	0.0072	61.2 (55.1–66.6)	48.3 (37.1–58.5)	0.011
HR (95% CI)	0.63 (0.45–0.88)	1 (ref)		0.71 (0.54–0.93)	1 (ref)	

^a^The response was assessed according to the local investigators per Lugano 2014 criteria, and the best response throughout patient follow-up was reported.

### Inverse probability of treatment weighting

**Extended Data Fig. 4:**
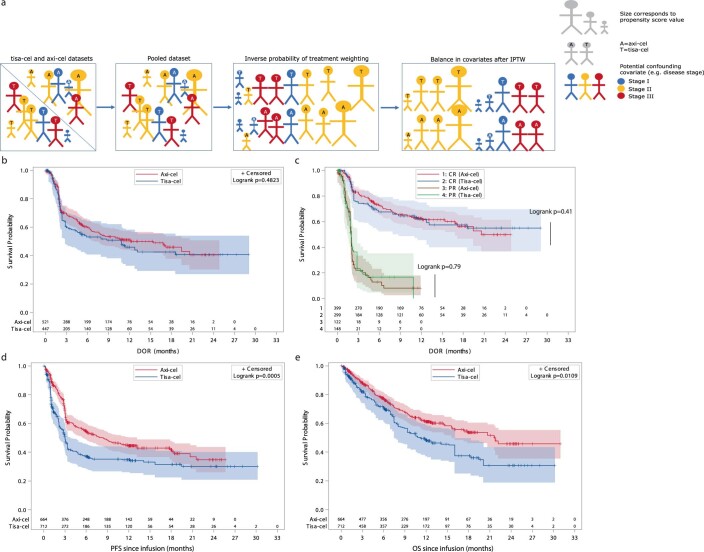
Response rates and survival according to CAR-T type after inverse probability of treatment weighting. **a**, In IPTW, weight of each individual patient is calculated as the inverse of their probability of receiving tisa-cel or axi-cel, assessed by their propensity score. Compared to PSM, it creates a pseudo-population of patients in which patients with a lower likelihood of receiving one CAR-T is over-weighted in the final population. As for PSM, comparability according to each covariate of the resulting 2 pseudo-cohorts of patients receiving one CAR-T or the other is checked using standardized mean differences (SMD, Extended Data Fig. 3). **b**, DOR according to CAR-T. **c**, DOR according to CAR-T and response quality. **d**, PFS according to CAR-T. **e**, OS according to CAR-T. Shaded areas correspond to the 95% confidence bands using Hall-Wellner method. *P* values were calculated using two-sided logrank tests. No adjustment was made for multiple comparisons.

Inverse probability of treatment weighting (IPTW) is another method where weights are assigned to patients based on the inverse probability of receiving one treatment or the other as estimated by the propensity score. IPTW results in a pseudo-population that is balanced regarding the distribution of patient covariates in each treatment group. After IPTW, absolute values of the SMDs were less than 0.1 for almost all matching covariates (Extended Data Fig. 3g,h). IPTW was used to support the findings of PSM analysis and to allow for proper comparison between the two populations of patients treated with axi-cel or tisa-cel. Using this statistical approach, significantly higher ORR/CRR and longer PFS and OS with axi-cel compared to tisa-cel were observed (Table 2 and Extended Data Fig. 4a–e). Of note, no difference was observed for DOR as in the PSM analysis.

### Safety in the propensity score matched populations

In the matched population (*n* = 418), 180 (86.1%) out of 209 patients treated with axi-cel experienced a cytokine release syndrome (CRS) of any grade compared to 158 (75.6%) patients out of 209 with tisa-cel (Table 3). Most CRSs were of grade 1 or 2 whatever the CAR T product. Grade 1–2 CRS was significantly more frequent with axi-cel than with tisa-cel (80.9% versus 66.5%; *P* < 0.001), but no significant difference was observed for grade ≥3 CRS (9.1% versus 5.3% for tisa-cel and axi-cel, respectively; *P* = 0.130).

**Table 3:** Toxicity after CAR T infusion according to CAR T product in the PSM cohorts

	axi-cel		tisa-cel		*P*
	*n* = 209		*n* = 209		
CRS of any grade	180	(86.1%)	158	(75.6%)	0.006
Grade 1–2	169	(80.9%)	139	(66.5%)	<0.001
Grade ≥3	11	(5.3%)	19	(9.1%)	0.130
ICANS of any grade	102	(48.8%)	46	(22.0%)	<0.001
Grade 1–2	73	(34.9%)	40	(19.1%)	<0.001
Grade ≥3	29	(13.9%)	6	(2.9%)	<0.001
Cytopenia of any grade at M1	135	(64.6%)	82	(39.2%)	<0.001
Grade 1–2	64	(30.6%)	56	(26.8%)	0.387
Grade ≥3	71	(34.0%)	26	(12.4%)	<0.001
Neutropenia of any grade at M1	124	(59.3%)	57	(27.3%)	<0.001
Grade 1–2	71	(34.0%)	37	(17.7%)	<0.001
Grade ≥3	53	(25.4%)	20	(9.6%)	<0.001
Anemia of any grade at M1	94	(45.0%)	58	(27.8%)	<0.001
Grade 1–2	90	(43.1%)	58	(27.8%)	0.001
Grade ≥3	4	(1.9%)	0	(0.0%)	0.044
Thrombocytopenia of any grade at M1	116	(55.5%)	62	(29.7%)	<0.001
Grade 1–2	70	(33.5%)	43	(20.6%)	0.003
Grade ≥3	46	(22.0%)	19	(9.1%)	<0.001
Cytopenia of any grade at M3	75	(35.9%)	29	(13.9%)	<0.001
Grade 1–2	51	(24.4%)	21	(10.0%)	<0.001
Grade ≥3	24	(11.5%)	8	(3.8%)	0.003
Neutropenia of any grade at M3	62	(29.7%)	22	(10.5%)	<0.001
Grade 1–2	44	(21.1%)	16	(7.7%)	<0.001
Grade ≥3	18	(8.6%)	6	(2.9%)	0.012
Anemia of any grade at M3	52	(24.9%)	15	(7.2%)	<0.001
Grade 1–2	51	(24.4%)	13	(6.2%)	<0.001
Grade ≥3	1	(0.5%)	2	(1.0%)	0.562
Thrombocytopenia of any grade at M3	58	(27.8%)	20	(9.6%)	<0.001
Grade 1–2	40	(19.1%)	13	(7.7%)	<0.001
Grade ≥3	18	(8.6%)	4	(1.9%)	0.002

Toxicities were graded according to CTCAE version 5.0 for cytopenias and according to the consensus grading from the ASTCT for CRS and ICANS. Only patients who experienced at least grade ≥1 toxicity are reported in the table. M1, 1 month; M3, 3 months.

Regarding ICANS, both low-grade (that is, grade ≤2) and severe (that is, grade ≥3) ICANS were significantly more frequent with axi-cel than with tisa-cel (Table 3). Thirty-five percent of patients experienced grade 1–2 ICANS after axi-cel infusion compared to 19.1% after tisa-cel infusion (*P* < 0.001). Twenty-nine patients (13.9%) presented a grade ≥3 ICANS with axi-cel compared to only six (2.9%) with tisa-cel (*P* < 0.001).

Hematological toxicity was also significantly more frequent and more severe with axi-cel than with tisa-cel (Table 3). Any grade cytopenia at 1 month after CAR T infusion was observed in 64.6% of patients compared to 39.2% and grade ≥3 cytopenia in 34.0% compared to 12.4% with axi-cel and tisa-cel, respectively (Table 3). Significantly higher hematological toxicity after axi-cel infusion compared to after tisa-cel infusion was consistent across all hematological lineages (that is, neutropenia, anemia and thrombocytopenia; Table 3). The same held true for prolonged cytopenias observed at 3 months after CAR T infusion (Table 3). Notably, no significant difference in cytopenias was observed before lymphodepletion between patients treated with axi-cel or with tisa-cel, meaning that the observed higher hematological toxicity with axi-cel was not attributable to significant baseline differences.

No grade 5 CRS deemed related to axi-cel was noted compared to two with tisa-cel in the matched-population. One grade 5 ICANS was reported with axi-cel but none with tisa-cel. No other grade 5 adverse event directly associated with CAR T infusion was reported in the matched populations.

### Subgroup analyses

**Extended Data Fig. 5:**
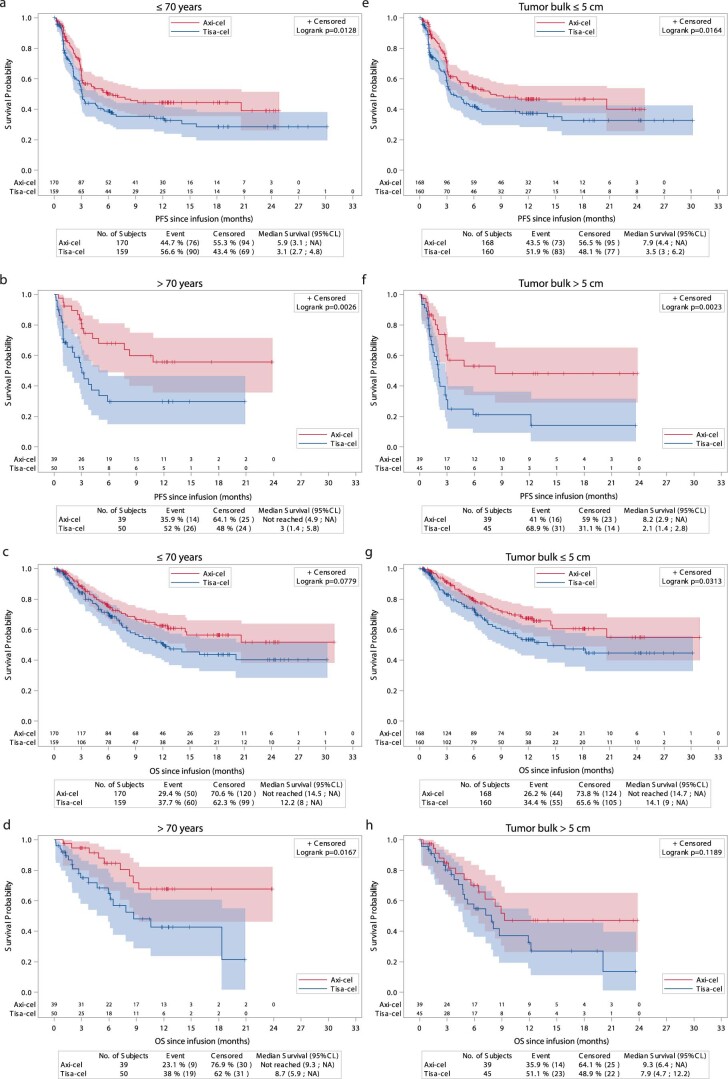
Planned subgroup analyses according to age and tumor bulk. **a**, PFS according to CAR T product in patients ≤ 70 years. **b**, PFS according to CAR T product in patients > 70 years. **c**, OS according to CAR T product in patients ≤ 70 years. **d**, OS according to CAR T product in patients > 70 years. **e**, PFS according to CAR T product in patients with ≤ 5 cm tumor bulk. **f**, PFS according to CAR T product in patients with > 5 cm tumor bulk. **g**, OS according to CAR T product in patients with ≤ 5 cm tumor bulk. **h**, OS according to CAR-T in patients with > 5 cm tumor bulk. Shaded areas correspond to the 95% confidence bands using Hall-Wellner method. P values were calculated using two-sided logrank tests. No adjustment was made for multiple comparisons.

Two subgroup analyses were originally planned. First, outcome according to age category (that is, ≤70 years and >70 years) was assessed in the PSM population. PFS was significantly longer after axi-cel infusion than after tisa-cel infusion both in patients aged 70 years or younger and in patients older than 70 years. Median PFS was 5.9 months compared to 3.1 months for axi-cel and tisa-cel, respectively, for patients ≤70 years (*P* = 0.0128) and was not reached compared to 3 months, respectively, for >70 years (*P* = 0.0026) (Extended Data Fig. 5a,b). For OS, survival was longer with axi-cel compared to tisa-cel in both age categories similarly, although statistical significance was not reached in patients ≤70 years (*P* = 0.0779 in the ≤70-years group and *P* = 0.0167 in the >70-years group, respectively) (Extended Data Fig. 5c,d). Second, because CAR T potency in the case of high tumor bulk could depend on the type of co-stimulatory domain, efficacy was evaluated according to the longest diameter of the largest node or extranodal mass taken as a correlate of the tumor bulk (that is, ≤5 cm or >5 cm). PFS was significantly longer regardless of tumor bulk after axi-cel infusion compared to after tisa-cel infusion. In the absence of a bulky mass, median PFS was 7.9 months with axi-cel and 3.5 months with tisa-cel (*P* = 0.0164). In the presence of a bulky disease, median PFS was 8.2 months with axi-cel and 2.1 months with tisa-cel (*P* = 0.0023) (Extended Data Fig. 5e,f). Better outcome with axi-cel than with tisa-cel regardless of tumor bulk held true for OS (Extended Data Fig. 5g,h).

### Sensitivity analyses

**Extended Data Fig. 6:**
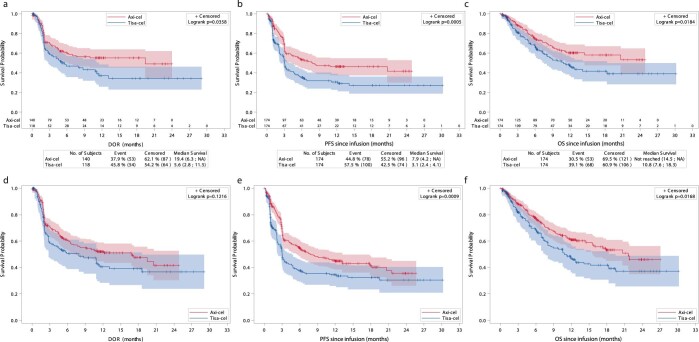
Survival according to CART product after PSM or IPTW in the complete case analysis (that is, where all cases with at least one missing value in matching covariates have been removed). **a**, DOR according to CART product after PSM. **b**, PFS according to CART product after PSM. **c**, OS according to CART product after PSM. **d**, DOR according to CART product after IPTW. **e**, PFS according to CART product after IPTW. **f**, OS according to CART product after IPTW. Shaded areas correspond to the 95% confidence bands using Hall-Wellner method. *P* values were calculated using two-sided logrank tests. No adjustment was made for multiple comparisons.

**Extended Data Fig. 7:**
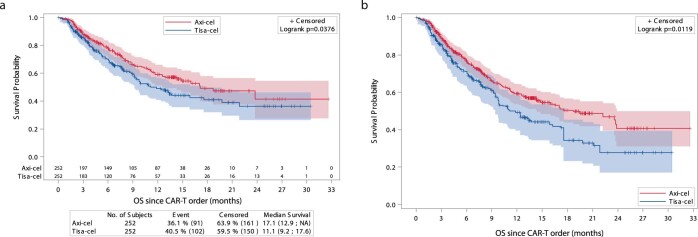
Overall survival from CAR-T order (instead of from infusion) according to CAR-T type after PSM or IPTW. **a**, OS according to CAR-T after PSM. **b**, OS according to CAR-T after IPTW. Shaded areas correspond to the 95% confidence bands using Hall-Wellner method. *P* values were calculated using two-sided logrank tests. No adjustment was made for multiple comparisons.

To ensure the robustness of comparison of results, several sensitivity analyses were performed. First, PSM and efficacy analyses were carried out on a subpopulation of patients with no missing data for any matching parameter. In total, 174 patients treated with axi-cel were 1:1 matched with 174 patients treated with tisa-cel (Extended Data Table 1). Similar results were found regarding ORR/CRR (Extended Data Table 2), DOR, PFS and OS (Extended Data Fig. 6a–f), with a superior efficacy of axi-cel compared to tisa-cel using both PSM and IPTW approaches. Apart from considering missing data as a category (missing indicator method) or from removing missing data (complete case analysis), multiple imputation approach on ten simulated datasets was also used and found similarly that patients treated with axi-cel experienced significantly prolonged PFS (HR = 0.64; 95% CI, 0.49–0.83) and OS (HR = 0.70; 95% CI, 0.51﻿–0.97) (Extended Data File 1). Furthermore, PSM and IPTW comparisons for OS were performed from CAR T order instead of CAR T infusion to avoid biases due to the manufacturing process. OS from CAR T order was significantly longer with axi-cel than with tisa-cel using both PSM or IPTW (*P* = 0.038 and *P* = 0.012, respectively; Extended Data Fig. 7a,b). Because a residual imbalance of adverse prognosis factors remained for patient treated with tisa-cel after stringent matching on 14 parameters, bivariate Cox analyses with CAR T product and aaIPI as explanatory variables were performed. Significantly prolonged PFS and OS were still associated with axi-cel compared to tisa-cel (HR = 0.64 and *P* = 0.012 for PFS and HR = 0.61 and *P* < 0.001 for OS, respectively).

Despite exhaustive matching on known and measured confounding factors, an unmeasured confounder can still lead to erroneous conclusions. For PFS and OS, the E-values were 2.18 (lower limit (LL) of the CI, 1.6) and 2.09 (LL of the CI, 1.39), respectively, meaning that the observed difference for PFS and OS between axi-cel and tisa-cel could be explained away only by an unmeasured confounder that was associated with both CAR T products and PFS (or OS) by a risk ratio of more than 2.18-fold each for PFS (or 2.09-fold each for OS).

## Discussion

In the present study, 809 patients for whom a CAR T order was obtained outside of a trial setting for DLBCL in second or subsequent relapse were analyzed. Median OS from CAR T order and CAR T infusion was 17 months and 19 months, respectively, for the whole cohort of patients. Strikingly, in the 1:1 matched population of 418 patients considered after the stringent PSM statistical approach, ORR/CRR were 66%/42% for tisa-cel and 80%/60% for axi-cel, which mirror response rates in the two pivotal clinical trials: JULIET and ZUMA-1 (52%/40% and 82%/58%, respectively)^5,6,8,9^. Similarly, median OS was 11.2 months with tisa-cel, whereas median OS was not reached with axi-cel, echoing the 11.1 months and 25.8 months of median OS in the recent updates of the JULIET and ZUMA-1 trials, respectively.

RWE studies are of utmost importance to assess if trial conclusions are reproducible in routine practice and if they can be applied to a more diverse patient population than the one strictly limited to pivotal trial enrollment criteria. Furthermore, RWE studies provide a critical basis from which to conduct cross-comparison analyses based on IPD. PSM and IPTW are increasingly used to address confounding by indication in RWE studies. The objectives of these statistical approaches are to balance out differences between patient groups that can be substantial and that preclude drawing firm conclusions when comparing outcome measurements. Subtle differences exist between the two methods that have been extensively reviewed elsewhere^25^. In the present study, both techniques similarly concluded that axi-cel provides better disease control than tisa-cel in R/R DLBCL after two lines of previous therapy.

After stringent matching to control for slightly more aggressive disease features in the patient population treated with tisa-cel (more frequent stage IV disease, older age and poorer performance status), ORR, CRR, PFS and OS were all significantly higher or longer after axi-cel infusion than after tisa-cel infusion. All sensitivity analyses, by considering time from CAR T order instead of time from infusion, by performing complete case analysis, by adjusting for residual imbalance in aaIPI or by using multiple imputation, led to the same exact conclusions.

Regarding toxicity, axi-cel was associated with significantly more frequent low-grade CRS and, more importantly, with significantly more frequent grade ≥3 ICANS. The rate of grade ≥3 ICANS reported here is low, with 13.9% and 2.9% for axi-cel and tisa-cel, respectively, in the matched population. In ZUMA-1, grade ≥3 ICANS was 31% for axi-cel and 12% for tisa-cel in the JULIET trial. In RWE for patients treated with axi-cel, Nastoupil et al.^12^ reported on 31% of grade ≥3 ICANS, whereas Jacobson et al.^11^ reported on 35%. Underreporting of severe neurotoxicity in the DESCAR-T registry cannot be excluded. However, it is well-known that new mitigation strategies for CRS and ICANS management have led to much lower rates of severe CRS or ICANS. For instance, recent data on prospective evaluation of early use of dexamethasone after axi-cel infusion demonstrated 17% of grade ≥3 ICANS^26^. In the study from the German group, grade ≥3 ICANS was 16% for axi-cel, quite similar to our data, and 7% for tisa-cel, slightly higher than what is reported here^27^. Moreover, marked and prolonged hematological toxicity was frequently observed after axi-cel infusion compared to after tisa-cel infusion. However, no significant difference was observed with regard to grade 5 adverse events. Therefore, even if higher efficacy with axi-cel comes at the cost of higher toxicity, the latter does not undermine the significantly better outcome. Because toxicity might be of greater concern in elderly patients and could counterbalance axi-cel’s higher efficacy, we undertook a planned subgroup analysis in patients aged 70 years and younger and those older than 70 years. Higher efficacy of axi-cel was still observed across age categories both for PFS and OS.

Interestingly, no significant difference was observed in DOR after PSM, whereas PFS was significantly longer with axi-cel. In fact, much of the PFS difference was related to the proportion of patients reaching a response after axi-cel as opposed to patients treated with tisa-cel and especially a complete response (60% versus 42% in the matched population). A 4-1BB-based autologous anti-CD19 CAR T product like tisa-cel is known to lead to longer persistence of the CAR T in vivo, but a CD28 co-stimulatory domain has been shown to lead to higher and faster proliferation^28,29^. Our findings provide strong clinical support to how these bio-cellular characteristics might translate into different disease controls. Recent data have suggested that a potential dose–response relationship exists between tumor burden before infusion and subsequent disease control with tisa-cel, suggesting that tisa-cel might be more potent in case of a lower tumor burden in DLBCL^30^. However, in a subgroup analysis, no difference in efficacy was further observed between tisa-cel and axi-cel in patients with or without a bulky disease at lymphodepletion assessed by a longest diameter of the largest node or mass >5 cm. Further correlations using total metabolic tumor volume (TMTV) or total lesion glycolysis (TLG), not readily available in DESCAR-T, will be of highest interest because it allows for a more accurate tumor bulk assessment.

Our study has limitations. First, most patient data were retrospectively collected. However, DESCAR-T is a monitored registry with high quality control. Second, a substantial amount of data were missing for PSM, and, if missing for important parameters, this may have allowed the introduction of significant residual uncontrolled bias. However, sensitivity analyses using a complete case analysis (instead of a missing indicator method), or using a multiple imputation approach, led to similar conclusions. Third, at only 11.7 months in the matched cohort, the median follow-up was short but was sufficient to reveal an OS difference between the two CAR T products. Recently reported survival curves with long follow-up of pivotal trials indicate that most deaths occur before 1 year after CAR T infusion, explaining why this short follow-up is sufficient to demonstrate significant statistical survival differences. Fourth, precise evaluation of HGBCL exhibiting double-hit or triple-hit chromosomic rearrangement by fluorescence in situ hybridization was not possible using histological data available in the registry and would require further queries or biomolecular testing. Nonetheless, almost all known confounding factors for efficacy after therapy with CAR T were taken into account in the PSM and ITPW approaches to ensure robust and balanced comparison between the two groups of patients treated with axi-cel or tisa-cel. Although the potential influence of unmeasured confounders may undermine the validity of causal conclusions, the magnitude of the observed outcome difference makes it unlikely as demonstrated by the high E-values above 2 found for both PFS and OS. This means that a relatively strong unmeasured confounding association (for instance, as strong as a poor performance status for which HR is 2.09 for PFS) would be needed to completely explain away the poorer outcome associated with tisa-cel.

At the end of last year, the ZUMA-7 randomized phase 3 trial comparing a standard of care (SOC) strategy (salvage regimen followed by ASCT) with axi-cel in second-line DLBCL demonstrated a significantly prolonged event-free survival (EFS) associated with axi-cel^31^. Conversely, the BELINDA trial, comparing tisa-cel to SOC in second-line DLBCL as well found no difference in EFS between the two randomized strategies^30^. Many design differences impaired a straight comparison between the two trials and their opposite conclusions. First, no bridging therapy was allowed before lymphodepletion in the ZUMA-7 trial except steroid use, as opposed to the BELINDA trial where bridging with chemotherapy was permitted. Second, early salvage regimen switching in the BELINDA trial was not considered an EFS event compared to the ZUMA-7 trial. Our RWE data suggest that, beyond extensive trial dissimilarities, a true efficacy difference between axi-cel and tisa-cel also probably substantiates the outcome divergence.

Furthermore, two RWE studies, partly addressing the same question using adjustment instead of matching, were recently reported^27,32^. In the first one, 356 patients treated with CAR T in Germany were considered (173 treated with axi-cel and 183 treated with tisa-cel). After adjusting for six parameters in a Cox model, PFS was significantly longer after axi-cel infusion than after tisa-cel infusion. No significant difference was observed for OS. In the second one, 68 patients from the United States treated with axi-cel were compared to 31 patients treated with tisa-cel, showing higher response rate after axi-cel infusion. With 809 patients analyzed, a comprehensive matching approach encompassing most of known confounding factors, multiple sensitivity analyses and a sufficient follow-up showing, to our knowledge for the first time, an OS advantage associated with axi-cel compared to tisa-cel, our study is one of the most mature to date.

In conclusion, although only a randomized study could allow for an undisputable comparison between the two CAR T products, our study is in favor of a higher efficacy but also a higher toxicity of axi-cel compared to tisa-cel in ≥3rd line of treatment for R/R DLBCL. These results need to be confirmed by other large RWE studies with similar statistical methods to account for imbalance between patient characteristics. Our findings could help in refining the choice of CAR T product for a specific patient based on the tradeoff between safety and efficacy.

## Methods

### Study design and patients

All patients treated in France with axi-cel or tisa-cel from December 2019 to October 2021 and retrospectively included in the DESCAR-T registry sponsored by the LYSARC were considered. Data export from the registry was set on 18 October 2021. All patients with DLBCL for whom a CAR T therapy with tisa-cel or axi-cel was ordered in the setting of the European Medicines Agency approval label (that is, after at least two prior lines of treatment) were considered. Patients could be treated (1) under French Temporary Authorization for Use (ATU); (2) under post-ATU authorization; or (3) under Market Authorization covered by the French health insurance system in an approved center. All patients received a non-opposition notice letter before enrollment, according to French laws. The protocol was approved by national ethics committees and the data protection agency, and the study was undertaken in accordance with the Declaration of Helsinki. DESCAR-T is registered under the ClinicalTrials.gov identifier NCT04328298.

### Outcomes

Primary outcome was PFS according to local investigator. Secondary outcomes were OS, best ORR and CRR, DOR and safety. Response was assessed according to the Lugano 2014 criteria, based on ^18^fluoro-deoxyglucose positron emission tomography (FDG-PET) at the approximate following timepoints: 1 month, 3 months, 6 months and 12 months after CAR T infusion^33^. Best response rate was considered. For all survival endpoints, survival was calculated from the date of CAR T infusion unless otherwise specified (that is, survival from CAR T order). PFS was defined from the date of CAR T infusion to the date of first documented relapse, progressive disease, date of last follow-up or death from any cause, whichever came first. OS was defined from the date of CAR T infusion or CAR T order to the date of death from any cause or the date of last follow-up. DOR was defined from the date of first response (partial or complete) to the date of first documented relapse, date of last follow-up or death from any cause, whichever came first. Hematological toxicity was graded according to the National Cancer Institute Common Terminology Criteria for Adverse Events (CTCAE, version 5.0). Hematological toxicity was reported in patients without initiation of a new treatment for progression or relapse after CAR T infusion. CRS and ICANS were graded according to the consensus criteria from the American Society for Transplantation and Cellular Therapy (ASTCT)^34^.

### Matching procedures

PSM was used to create a balanced covariate distribution between a cohort of patients treated with axi-cel and a cohort of patients treated with tisa-cel. Propensity scores were estimated using a multivariate logistic regression model with CAR T type (axi-cel versus tisa-cel) as the dependent variable. An exhaustive list of covariates was used for PSM: age (as a continuous parameter), sex, lactate dehydrogenase (LDH) level (normal versus between the upper limit of normal (ULN) and 2× ULN versus >2× ULN), C reactive protein (CRP) (dichotomized with a cutoff set at 30 mg L^−1^), time between last treatment and infusion (continuous), Eastern Cooperative Oncology Group (ECOG) performance status (PS) (0﻿–1 or ≥2), Ann Arbor stage (I versus II versus III versus IV), number of prior lines of treatment before CAR T (2–4 versus >4), bridging and response to bridging (no bridge versus bridging and response (partial or complete) to bridging versus bridging and no response (stable or progressive disease)), prior SCT either autologous or allogeneic (yes versus no), bulk assessed at lymphodepletion (dichotomized with a cutoff set at 5 cm), center (all centers with fewer than 20 patients were grouped into one category) and diagnosis (DLBCL NOS or HGBCL versus transformed indolent lymphoma (tFL or tMZL)). To account for a given center experience in CAR T procedure implementation and improvement of toxicity management over time that might impact outcome (especially because some centers had access to one CAR T before the other), time between first CAR T order for that center and CAR T infusion for a given patient was also considered for PSM (as a continuous parameter). For all matching parameters except continuous variables (no missing value could be used for continuous parameters in PSM), missing data were considered as one distinct category for PSM. Of note, when survival was assessed from CAR T order instead of CAR T infusion, time intervals were calculated until or from CAR T order instead of CAR T infusion. Matching parameters are detailed in Extended Data Table 3.

Matching was performed considering a 1:1 ratio without replacement and with optimal matching applying a caliper width of the propensity score set at 0.1. Basically, a patient treated with tisa-cel was selected and then matched with a patient treated with axi-cel given the constraint that the difference between the logit (that is, the logarithm of the odds of the logistic regression that models the probability of receiving tisa-cel or axi-cel) was less than a pre-specified maximum (that is, the caliper distance).

IPTW was used as another statistical approach to allow for outcome comparison between patients treated with axi-cel and patients treated with tisa-cel. In the IPTW method, the weight for each patient is calculated by inverting the probability of receiving the treatment the patient actually receives. PSM and IPTW rely on different statistical matching approaches, provide different information and should be interpreted differently. The first one (PSM) allows for assessing average treatment effect for the treated (ATT), whereas the other (IPTW with the weighting technique used here) provides estimation of the average treatment effect (ATE). The first gives the average effect of treatment on those patients who ultimately received one CAR T versus the other, whereas the second provides the average effect of theoretically moving the entire population from receiving one CAR T to the other. For IPTW, the exact same covariates as for PSM were used for the logistic regression model to calculate the propensity of receiving one of the CAR T products versus the other. Methodology underlying propensity-score-based matched comparisons and differences with adjustment approaches have been reviewed elsewhere^35^.

### Sensitivity analyses

Several sensitivity analyses were conducted. First, all patients with at least one missing value for at least one matching variable were removed from PSM analysis (complete case analysis). Second, a multiple imputation approach was performed using the fully conditional specification (FCS) method, allowing for different distributions across variables. Continuous variables were imputed using linear regression, whereas categorical parameters were imputed using logistic regression. All propensity score covariates and outcome (OS) were used for imputation. Ten imputed datasets were generated. A treatment effect was estimated within each imputed dataset using PSM. Estimated treatment effects from each imputed dataset were then combined into a single treatment effect using Rubin’s rule (within method). Third, a Cox bivariate model adjusting for residual aaIPI imbalance after matching was used to assess association between CAR T product and outcome (PFS and OS). Fourth, PSM was performed with a time of origin for OS set at the time of CAR T order instead of the time of CAR T infusion. Finally, to assess how robust the association between CAR T product and outcome was to potential unmeasured or uncontrolled confounding, E-value was computed^36^. It represents the minimum strength of association that a unique (or a set of) unmeasured confounder would need to have with both the treatment and the outcome conditional on the measured covariates to fully explain away the association between treatment (here, the CAR T product) and the outcome (here, PFS or OS). Therefore, the higher the E-value, the stronger the confounder associations must be to explain away the effect.

### Statistical analysis

Survival distributions were compared using the log-rank test. Response rates were compared using the χ^2^ test. A two-sided *P* value of less than 0.05 was considered significant. No adjustment was performed for multiple testing. Two subgroup analyses according to age (≤70 years and >70 years) and tumor bulk (≤5 cm and >5 cm) were pre-planned in the statistical analysis plan. Survival curves were generated using the Kaplan–Meier estimation method. Statistical analyses were performed using SAS software version 9.3 and R version 4.2.0. The MATCH macro for PSM and the MI and MIANALYZE procedures for multiple imputation were used with SAS. The E-value package was used with R.

### Reporting summary

Further information on research design is available in the Nature Research Reporting Summary linked to this article.

## Online content

Any methods, additional references, Nature Research reporting summaries, source data, extended data, supplementary information, acknowledgements, peer review information; details of author contributions and competing interests; and statements of data and code availability are available at 10.1038/s41591-022-01969-y.

### Supplementary information

Reporting Summary

Supplementary Table 1 A multiple imputation approach was performed using FCS, allowing for different distributions across variables. Continuous variables were imputed using linear regression, whereas categorical parameters were imputed using logistic regression. All propensity score covariates and outcome (OS) were used for imputation. Ten imputed datasets were generated. A treatment effect was estimated within each imputed dataset using PSM. Estimated treatment effects from each imputed dataset were then combined into a single treatment effect using Rubin’s rule (within method).

## Figures and Tables

**Extended Data Table 1:**
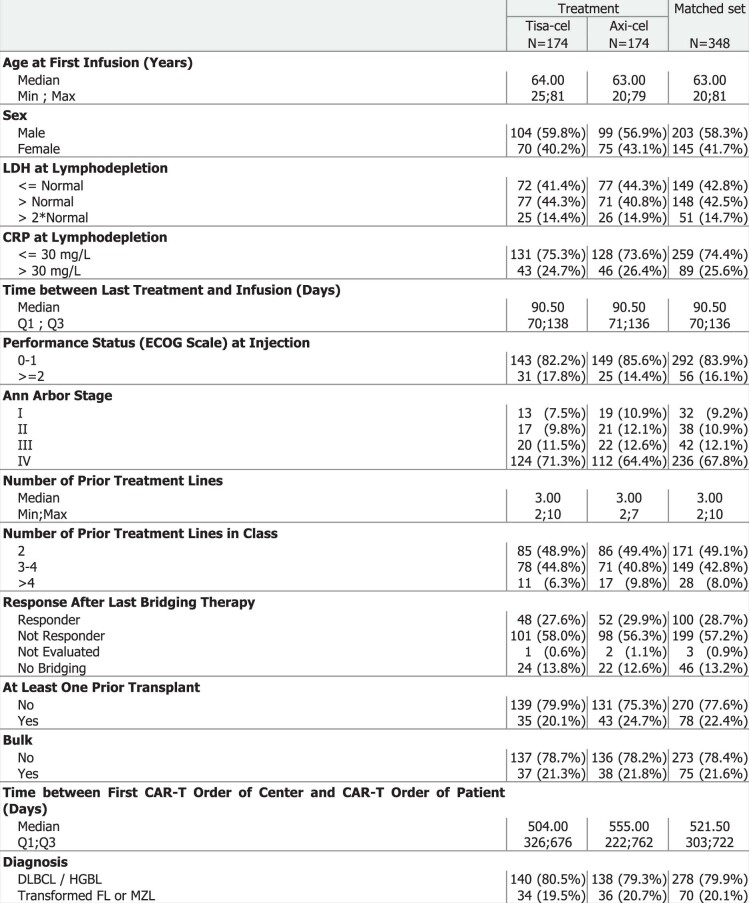
Patient characteristics in the matched cohorts without any missing data for matching covariates (that is, complete case analysis)

**Extended Data Table 2:**
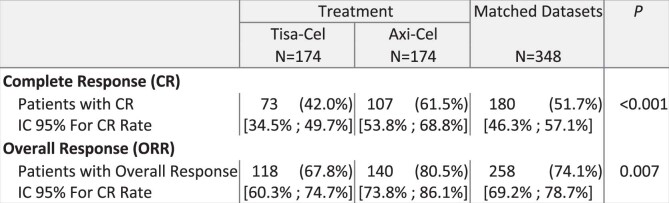
Response rates in the matched cohorts without any missing data for matching covariates. Patients without response assessment (due to whatever reason) are considered as non-responders

**Extended Data Table 3:**
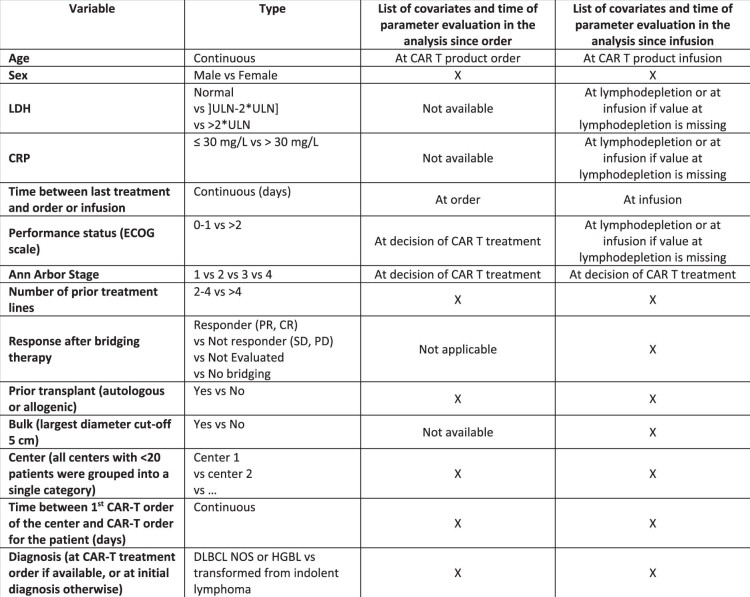
List of covariates used for propensity score calculation

## Data Availability

Data from the DESCAR-T registry are subject to controlled access by the LYSARC owing to privacy and legal requirements and to proprietary reasons. Anonymized IPD requests will be promptly reviewed by the corresponding author (E.B.) and the scientific committee of the DESCAR-T registry. Individual de-identified participant data will be made available for replication and validation purposes from the present study only. For any other reason, an agreement for data sharing will depend on the nature of the request, the intended use of the data and their availability, as well as the merit of the research project. Agreement will be made after the DESCAR-T scientific committee decision, and a data sharing agreement will have to be signed before any data transfer. All requests should be addressed to descar-t@lysarc.org. A reply will be provided within 1 month after the data request.
