# Supplementary material for: A real-world comparison of tisagenlecleucel and axicabtagene ciloleucel CAR T cells in relapsed or refractory diffuse large B cell lymphoma
Source: Nat Med. 2022 Sep 22;28(10):2145–54. doi: 10.1038/s41591-022-01969-y (PMC9556323; doi:10.1038/s41591-022-01969-y)
Supplement: Supplementary file 1 — Reporting Summary [file 41591_2022_1969_MOESM1_ESM.pdf]

nature portfolio

Corresponding author(s): Prof. Emmanuel BACHY, M.D. Ph.D.

Last updated by author(s): Mar 28, 2022

Reporting Summary

Nature Portfolio wishes to improve the reproducibility of the work that we publish. This form provides structure for consistency and transparency in reporting. For further information on Nature Portfolio policies, see our Editorial Policies and the Editorial Policy Checklist.

Statistics

For all statistical analyses, confirm that the following items are present in the figure legend, table legend, main text, or Methods section.

n/a	Confirmed	
[x]	[ ]	The exact sample size (*n*) for each experimental group/condition, given as a discrete number and unit of measurement
[x]	[ ]	A statement on whether measurements were taken from distinct samples or whether the same sample was measured repeatedly
[ ]	[x]	The statistical test(s) used AND whether they are one- or two-sided<br>*Only common tests should be described solely by name; describe more complex techniques in the Methods section.*
[ ]	[x]	A description of all covariates tested
[x]	[ ]	A description of any assumptions or corrections, such as tests of normality and adjustment for multiple comparisons
[ ]	[x]	A full description of the statistical parameters including central tendency (e.g. means) or other basic estimates (e.g. regression coefficient) AND variation (e.g. standard deviation) or associated estimates of uncertainty (e.g. confidence intervals)
[ ]	[x]	For null hypothesis testing, the test statistic (e.g. *F*, *t*, *r*) with confidence intervals, effect sizes, degrees of freedom and *P* value noted<br>*Give P values as exact values whenever suitable.*
[x]	[ ]	For Bayesian analysis, information on the choice of priors and Markov chain Monte Carlo settings
[x]	[ ]	For hierarchical and complex designs, identification of the appropriate level for tests and full reporting of outcomes
[x]	[ ]	Estimates of effect sizes (e.g. Cohen's *d*, Pearson's *r*), indicating how they were calculated

Our web collection on statistics for biologists contains articles on many of the points above.

Software and code

Policy information about availability of computer code

Data collection	EDC system (Electonic Data Capture from Ennov group) version 8.1
Data analysis	SAS v9.3

For manuscripts utilizing custom algorithms or software that are central to the research but not yet described in published literature, software must be made available to editors and reviewers. We strongly encourage code deposition in a community repository (e.g. GitHub). See the Nature Portfolio guidelines for submitting code & software for further information.

Data

Policy information about availability of data

All manuscripts must include a data availability statement. This statement should provide the following information, where applicable:

- Accession codes, unique identifiers, or web links for publicly available datasets
- A description of any restrictions on data availability
- For clinical datasets or third party data, please ensure that the statement adheres to our policy

Data from the DESCAR-T registry are subject to controlled access by the LYSARC due to privacy and legal requirement and to proprietary reasons. Anonymized individual patient data (IPD) request will be promptly reviewed by the corresponding author (EB) and the scientific committee of the DESCAR-T registry. Individual de-identified participant data will be made available for replication and validation purpose of results from the present study only. For any other reason, agreement for data sharing will depend on the nature of the request, the intended use of the data and their availability, as well as the merit of the research project. Agreement will be made following the DESCAR-T scientific committee decision and a data sharing agreement will have to be signed before any data transfer. All requests should be addressed to descar-t@lysarc.org. Reply will be provided within one month following data request.

Field-specific reporting

Please select the one below that is the best fit for your research. If you are not sure, read the appropriate sections before making your selection.

☒ Life sciences ☐ Behavioural & social sciences ☐ Ecological, evolutionary & environmental sciences

For a reference copy of the document with all sections, see nature.com/documents/nr-reporting-summary-flat.pdf

Life sciences study design

All studies must disclose on these points even when the disclosure is negative.

Sample size	No sample size calculation was required for this retrospective analysis with no statistical assumption a priori. All patients included in the French DESCAR-T registry were analyzed in the study.
Data exclusions	No data from patients with DLBCL treated in ≥3rd line of treatment and part of the DESCAR-T registry were excluded (see patients flow).
Replication	Does not apply to clinical patient data included in the study. Many sensitivity analyses were performed to ensure robustness of the results with missing data indicator category, complete case analysis, analyses from CAR-T order instead of infusion, multiple imputation approaches and unmeasured confounder evaluation.
Randomization	No randomization was considered in this retrospective study. All potential measured confounders were taken into consideration using propensity score matching and inverse probability of treatment weighting. An extensive list of fourteen covariates were used for matching.
Blinding	No blinding was performed in the study since commercial CAR-T product order and infusion in routine practice was analyzed (real-worl evidence data).

Reporting for specific materials, systems and methods

We require information from authors about some types of materials, experimental systems and methods used in many studies. Here, indicate whether each material, system or method listed is relevant to your study. If you are not sure if a list item applies to your research, read the appropriate section before selecting a response.

Materials & experimental systems

n/a	Involved in the study	
☒	☐	Antibodies
☒	☐	Eukaryotic cell lines
☒	☐	Palaeontology and archaeology
☒	☐	Animals and other organisms
☐	☒	Human research participants
☐	☒	Clinical data
☒	☐	Dual use research of concern

Methods

n/a	Involved in the study	
☒	☐	ChIP-seq
☒	☐	Flow cytometry
☒	☐	MRI-based neuroimaging

Human research participants

Policy information about studies involving human research participants

Population characteristics	All patient characteristics are detailed in Table 1 of the manuscript
Recruitment	All patients treated in France with axi-cel or tisa-cel from December 2019 to October 2021 and retrospectively included in the DESCAR-T registry sponsored by LYSARC were considered. Data export from the registry was set on the 18TH of October 2021. All patients with DLBCL for whom a CAR-T therapy with tisa-cel or axi-cel was ordered in the setting of the European Medical Agency (EMA) approval label (i.e., after at least 2 prior lines of treatment) were considered.
Ethics oversight	The protocol was approved by national ethics committees and the data protection agency from France, and the study was undertaken in accordance with the Declaration of Helsinki.

Note that full information on the approval of the study protocol must also be provided in the manuscript.

Clinical data

Policy information about clinical studies

All manuscripts should comply with the ICMJE guidelines for publication of clinical research and a completed CONSORT checklist must be included with all submissions.

Clinical trial registration	DESCAR-T is registered under the ClinicalTrials.gov identifier NCT04328298.
Study protocol	No specific protocol for planned ancillary study based on the retrospective DESCAR-T registry is available.
Data collection	All patients treated in France with axi-cel or tisa-cel from December 2019 to October 2021 and retrospectively included in the DESCAR-T registry sponsored by LYSARC were considered. Data export from the registry was set on the 18TH of October 2021. A full list of participating centers can be found on the ClinicalTrial.gov website: https://clinicaltrials.gov/ct2/show/NCT04328298
Outcomes	Primary outcome was PFS according to local investigator. Secondary outcomes were overall survival OS, best ORR and CRR (according to Lugano 2014 criteria), DOR and safety. All information can be retrieved from the Patients & Methods section of the manuscript.
